# Validating Machine Learning Algorithms for Twitter Data Against Established Measures of Suicidality

**DOI:** 10.2196/mental.4822

**Published:** 2016-05-16

**Authors:** Scott R Braithwaite, Christophe Giraud-Carrier, Josh West, Michael D Barnes, Carl Lee Hanson

**Affiliations:** ^1^ Computational Health Science Research Group Department of Psychology Brigham Young University Provo, UT United States; ^2^ Computational Health Science Research Group Department of Computer Science Brigham Young University Provo, UT United States; ^3^ Computational Health Science Research Group Department of Health Science Brigham Young University Provo, UT United States

**Keywords:** suicide, social media, twitter, machine learning

## Abstract

**Background:**

One of the leading causes of death in the United States (US) is suicide and new methods of assessment are needed to track its risk in real time.

**Objective:**

Our objective is to validate the use of machine learning algorithms for Twitter data against empirically validated measures of suicidality in the US population.

**Methods:**

Using a machine learning algorithm, the Twitter feeds of 135 Mechanical Turk (MTurk) participants were compared with validated, self-report measures of suicide risk.

**Results:**

Our findings show that people who are at high suicidal risk can be easily differentiated from those who are not by machine learning algorithms, which accurately identify the clinically significant suicidal rate in 92% of cases (sensitivity: 53%, specificity: 97%, positive predictive value: 75%, negative predictive value: 93%).

**Conclusions:**

Machine learning algorithms are efficient in differentiating people who are at a suicidal risk from those who are not. Evidence for suicidality can be measured in nonclinical populations using social media data.

## Introduction

Suicide claims more than twice as many lives each year as compared with homicide, and is the 10th leading cause of death in the United States (US). It is found that the suicide count rises above 33,000 every year [1] and more than 30 people attempt suicide following each death [1]. This eventually results in emotional and financial burdens on their families and loved ones. The World Health Organization [2] recently endorsed several universal interventions to prevent suicide, of which two promising strategies were to target vulnerable groups and individuals and then facilitate their access to crisis helplines. Timely identification of these vulnerable groups and individuals [3], and the balance of identifying high-risk cases without too many false positives [4,5], however, remains a challenge. This has led to increasing efforts in clinical settings, which further increase financial and time-related costs [6]. Due to these reasons, the public health priority is to explore novel approaches and identify individuals at risk for suicide without increasing costs or adding burdens to the already present clinical system. This effort may benefit from the introduction and proliferation of emerging social media technologies.

Social media has provided researchers with new avenues to employ automated methods for analyzing language and to better understand individuals’ thoughts, feelings, beliefs, behavior, and personalities [7]. Studies of language-using computational data-driven methodologies have demonstrated utility for monitoring psychological states and public health problems such as influenza [8,9], heart disease mortality [10], drinking problem [11], prescription drug abuse [12,13], and tobacco use [14]. Infodemiology or infoveillance is an emerging field related to computational data-driven methodologies and other studies, that use social media to understand and monitor health problems efficiently [15,16].

Twitter is a social media application that allows users to broadcast news, information, and personal updates to other users (followers) in tweets or statements of 140 characters or less. Speech is considered to be an important marker for both depression and suicide risk assessment [17], and Twitter provides a novel social media avenue for exploring these major public health concerns. Initial studies confirmed the role of infodemiology or infoveillance for social media data to track health trends [8-14], something beyond its original purpose. However, these early studies are limited as they failed to prove that the observed trends reflect the actual values. The advanced research in several studies has focused on confirming social media observations, which increases our collective confidence in these data values that act as a source for monitoring health issues and trends [18-20]. Most relevant to the work presented here, recent studies have used Twitter, Sina Weibo (Sina Weibo is a Chinese social media and microblogging site similar to Twitter), and Reddit specifically to tackle suicidality [21-25]. An annual workshop series commenced in 2014 on *Computational Linguistics and Clinical Psychology: From Linguistic Signal to Clinical Reality*, has attracted a host of social media data-driven work on suicide, as well as other mental health issues, including depression, schizophrenia, dementia, and posttraumatic syndrome disorder (eg, see [26-30]). Additional research remains warranted to demonstrate the safety and efficacy of social media prevention activities [31], and methodological issues need further refinement especially in terms of specificity and sensitivity of suicide risk. The purpose of this study was to validate the use of machine learning for Twitter data against empirically validated measures of suicidality in the US population with an eye for suicide prevention.

## Methods

### Recruitment and Procedure

The participants for this study were selected through Amazon’s Mechanical Turk (MTurk, www.mturk.com). Participants in the US who were frequent Twitter users and above 18 years of age were invited to participate in a “Survey for Twitter users (~10 min).” Only those who had completed more than 500 Human Intelligence Tasks (HITs)—the name MTurk gives to online tasks, including surveys, transcriptions, categorization of receipt items, etc.—with an approval rate of > 95% (ie, requesters found their work was acceptable for more than 95% of tasks they had undertaken) were allowed to complete the survey. Participants were informed that this survey was for Twitter users and that “Only those who are active Twitter users with public, personal Twitter accounts may participate, we will not approve any workers who do not meet these qualifications.” To ensure the eligibility of participants, they had to complete a screening questionnaire before accepting the HIT. The screening questionnaire questioned whether they had an active, public Twitter account, how long the account had been active, and how often they tweeted. Our survey was published during the early summer of 2014 and republished during the early fall of 2015. Participants were paid according to the current MTurk market rates (ie, between 30 and 50 cents). The authors’ university institutional review board approved all the study procedures and measures.

### Stimuli: Human Intelligence Tasks

Participation in the study consisted of providing a Twitter handle and completing a set of questionnaires that assessed psychosocial functioning. A Twitter handle is a username that takes the form @username. The questionnaires examined in the present study are the Depressive Symptom Inventory–Suicide Subscale (DSI-SS), The Interpersonal Needs Questionnaire (INQ), and Acquired Capability for Suicide Scale (ACSS). The DSI-SS, a 4-item screening tool for suicidal symptoms assesses suicidality in a reliable and valid manner. In addition to an established clinical cutoff, it assesses for resolved plans and presuicidal actions, which are absent in most suicidal cases [32]. The INQ and ACSS scales assess facets of Joiner’s Interpersonal Theory of suicide: thwarted belongingness, perceived burdensomeness (INQ), and the acquired capability for suicide (ACSS) [33]. These scales have demonstrated good reliability and construct validity [34].

### Participants

In Summer 2014, we decided to obtain a sample of 100 participants. Beginning with 489 potential participants, we dropped 251 participants that did not actually provide data (most of these were likely bots, which are computer programs designed to generate responses to HITs in hopes of receiving payment). Researchers studying MTurk data collection recommended involving high reputation participants (those with a high number of completed and approved HITs) and including attention control checks [35]. We included control questions to ensure that those who responded were providing reliable data. Our control questions were designed to discern whether the participant was paying attention to each question (eg, “In the last month how often have you showed that you were paying attention by selecting ‘Sometimes’”). We included five control questions; participants who failed two or more were excluded. About 46 participants who failed to answer the control questions were excluded. Finally, five participants attempted the survey more than once, in some cases with variable answers. Since, it was impossible to decide which of their answers was valid, we removed these respondents and their duplicates (17 participants in total), resulting in 175 participants. To validate the Twitter handles provided by the corresponding MTurk participants, we used the Twitter API via Twitter4J, and queried the last status by the handle. We removed all users who could not answer the query as it indicates that either the user does not exist or the user’s account is not public. As individuals sometimes find it funny to mention a celebrity handle as their own, we verified that the user was not a celebrity. If an account was verified as a celebrity account, we removed the corresponding user. In addition, to ensure that the MTurk participants had some activity on Twitter and that they would have a sufficient number of tweets for our analysis, we removed all users who posted less than two posts per month on an average. To find the average number of posts per month, we divided the total number of tweets that the user posted by the number of months, since the user’s account was created. Finally, we removed all users whose last tweet was more than 1 month old. In total, there were 101 MTurk workers with both permissible responses and exploitable Twitter accounts. In Summer and Fall 2015, we repeated the above data collection procedure to extend the size of our sample. We began with 111 more potential candidates, excluded 77 of them, resulting in an additional 34 exploitable Twitter accounts for a final sample of size *N*=135. Participant characteristics are shown in Table 1. For all valid user accounts, we again queried the Twitter API to collect the latest 200 tweets of the user.

**Table 1:** Participant characteristics

Ethnicity	
African American	19 (14.1%)
Asian	5 (3.7%)
Latino	6 (4.4%)
Mixed/Biracial	9 (6.7%)
Caucasian (White)	95 (70.4%)
Native American	1 (0.7%)
Education	
Graduate of professional degree	14 (10.4%)
Bachelor’s degree	48 (35.6%)
Some college	60 (44.4%)
High school or equivalent (eg, GED)	9 (6.7%)
Less than high school	4 (2.9%)
Income	
Over $150K	2 (1.5%)
$100K–$150K	4 (3.0%)
$75K–$100K	12 (8.9%)
$50K–$75K	28 (20.7%)
$25K–$50K	41 (30.3%)
Under $25K	46 (34.1%)
None	2 (1.5%)
Twitter account creation date	
2008	14 (10.4%)
2009	39 (28.9%)
2010	17 (12.6%)
2011	23 (17.1%)
2012	20 (14.8%)
2013	13 (9.6%)
2014	8 (5.9%)
2015	1 (0.7%)

^a^GED General Educational Development

### Analysis of Tweets

For each participant, the textual content of all of the retrieved tweets was aggregated into a single file. Each file was then analyzed with the updated 2015 version of Linguistic Inquiry and Word Count software (LIWC) [36]. LIWC is a language analysis tool that extracts information from text in three main forms. The first, new in the 2015 version, consists of four variables that capture global high-level properties of the text as percentiles, namely, analytical thinking, clout, authenticity, and emotional tone. The second consists of 71 variables that represent the relative use of predefined word categories, from linguistic forms, such as pronouns and verbs, to psychological, social, emotional, and cognitive mechanisms, such as family, anger, sadness, certainty, leisure, religion, and death. The third focuses on the relative use of 17 language markers (eg, swear words, netspeak) and punctuation categories. For each of its 88 base categories, LIWC computes the percentage of total words in that category within the body of text being analyzed. For example, if a text sample has 125 words, and 3 of these words belong the pronoun category, LIWC gives a score of 2.4 (3/125) to that category. LIWC has been validated in a number of studies in the context of social media data [37,38] and has been previously proved to correlate in meaningful ways with suicidality [39-41]. LIWC has also been used to annotate tweets as showing signs of distress, where distress is regarded as a risk factor for suicide [42], as well as to analyze language differences across ten mental health issues [43].

### Description of Sample

Our sample consists of 85 females and 50 males with an ethnic composition largely consistent with the US population, with a slight under-representation of Latino, and overrepresentation of mixed/biracial individuals. The distribution level of education and income suggests that our sample consists of generally more educated and affluent individuals than the national average, consistent with the findings of other researchers [44]. About 7 (5.2%) individuals were identified as homosexual, 18 (13.3%) as bisexual, and 110 (81.5%) as heterosexual. The proportion of individuals who were not heterosexual is higher than that expected from population norms, perhaps because social media provides readier access to lesbian, gay, bisexual, and transgender (LGBT) populations than traditional methods of sampling [45]. All Twitter accounts were listed as English accounts, and most users had been active for several years as shown by the distribution of account creation dates in Table 1. Almost half of the users (64 of 135) had posted over 2000 tweets at the time of data collection. About 17 individuals in our sample could be confidently considered as clinically significantly suicidal, since their DSI-SS score was greater than 2; the remaining 118 individuals were deemed nonsuicidal.

### Machine Learning

For each participant, we built a feature vector consisting of the LIWC variables, together with a target class label: suicidal, nonsuicidal (as determined by the DSI-SS). The set of 135 vectors form a training data set that can be used by classification learning algorithms to induce a predictive model of suicidality. We implemented the predictive analysis in Python, using the scikit-learn library [46].

Various classification learning algorithms are available. For this study, our aim was to build not only a model with good accuracy but also one that would potentially provide insight into its predictions. Hence, decision tree learning was selected as it empirically produced accurate models for a large number of applications, and the models it built are easily interpretable [47]. Decision tree learning implements a kind of divide-and-conquer approach to learning. At each stage, a feature is selected and becomes the root of a subtree whose branches are the values, or ranges of values, of the selected feature. The training data are partitioned along these values, and sent down the corresponding branch. The process is then applied recursively to each partition until all training examples in the partition have the same label or there are no more features to select; at this point, a leaf node is created and labeled with the most prevalent label in the partition. A new example is classified by starting at the root of the tree, and following a path to a leaf node such that at each internal node the example takes the branch corresponding to its value for the feature at that node. The leaf node label is the predicted label for the new example. Note that the prevalence computed for a leaf node during training may in turn serve as a measure of confidence in its predictions. During learning, feature selection is affected by maximizing gain of information or the difference between the entropy of the training data before and after partitioning. Entropy measures the purity of a set of training examples with respect to the class label. A set where all of the examples have the same label has minimum entropy, while a set where the examples are spread uniformly over all labels has maximum entropy. Hence, at each stage, the attribute that is best at discriminating among the training examples at that stage is selected.

We estimate the accuracy of our decision tree learning approach using leave-one-out cross-validation (loo-cv), wherein, a decision tree is induced from all but one of the participants’ feature vectors and tested on the out-of-training participant. The process is repeated *N* times, until each participant has been left out for testing. For each participant, we record whether the prediction was correct and aggregate over all participants to obtain an overall accuracy value.

## Results

Analysis of responses to the INQ across the entire group and for each subgroup (suicidal and nonsuicidal, as defined by the DSI-SS cut-point) revealed significant differences. Suicidal individuals endorsed significantly less belongingness (one-tailed independent sample *t* (133) = -5.84, *p*<.001; Cohen’s *d*=-1.52, 95% *CI* [-2.05, -0.97]), and significantly higher burdensomeness (*t* (133) = -8.41, *p*<.001; *d*=-2.18, 95% *CI* [-2.75, -1.61]). Those indicating significant suicidality reported a slightly higher acquired capability for suicide (*t* (133) = -1.91, *p*=.03; *d*=-0.49, 95% *CI* [-1.00, 0.19]). These results offer additional support for the validity of the INQ and provide converging evidence of the suicidality of those who were above the cutoff on the DSI-SS.

If the DSI-SS cutoff identifies 17 individuals in our sample as suicidal, then the default accuracy of a predictive model, obtained by indiscriminately predicting the most prevalent class (here, nonsuicidal), is 87.4% (118/135). The decision tree’s loo-cv accuracy was 91.9%. The confusion matrix, shown in Table 2 , gives rise to the following values.

**Table 2:** Loo-cv confusion matrix for decision tree learning

	Suicidal	Not suicidal
Suicidal	9	8
Not suicidal	3	115

- Sensitivity: 0.53 (8 suicidal individuals were wrongly labeled as nonsuicidal)
- Specificity: 0.97 (only 3 out of 118 nonsuicidal individuals were wrongly labeled as suicidal)
- Positive predictive value: 0.75 (only 3 of the 12 individuals labeled as suicidal were actually not suicidal)
- Negative predictive value: 0.93 (only 8 of the 123 individuals labeled as nonsuicidal were actually suicidal)

**Figure 1:**
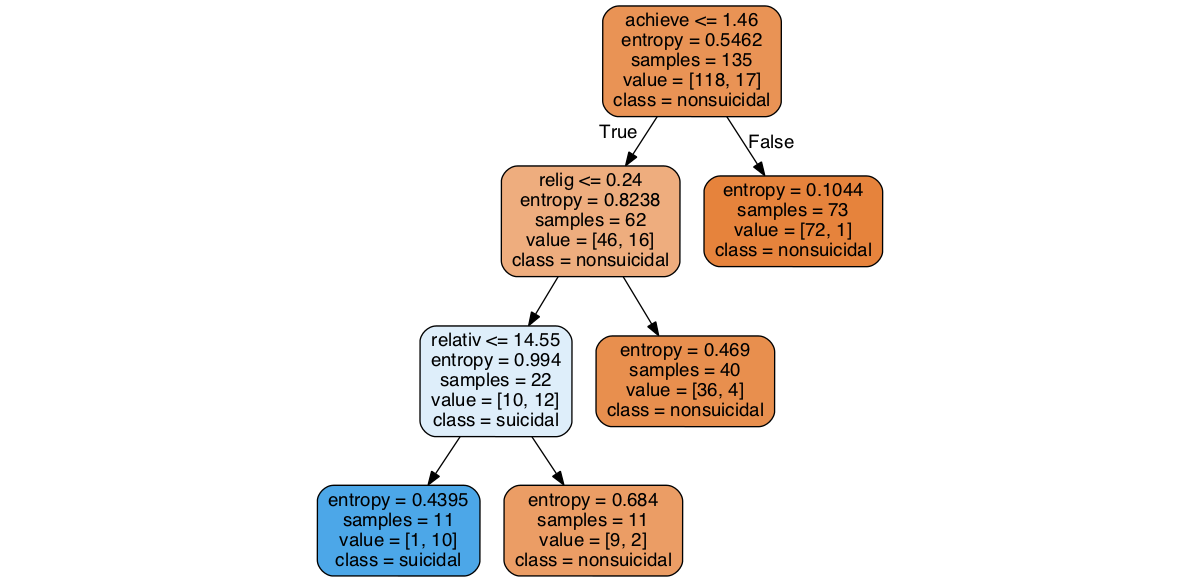
Result from Decision Tree Learning Algorithm.

The pruned decision tree induced from the complete sample is shown in Figure 1. It is included here for its explanatory power.

We note that there were minor differences in deeper parts of the unpruned trees induced over various runs, as increased depth tends to lead to overfitting. However, the macrostructure of the pruned tree depicted in Figure 1 remains consistent across runs, suggesting that the results should generalize.

The structure of the tree is rather consistent with intuition as well. The tree first splits on the “achieve” category of LIWC, such that if an individual’s usage rate of achievement-related words exceeds 1.46, that individual is labeled as nonsuicidal. It is striking that the corresponding leaf node has very low entropy, indicating that 72 of the 73 individuals in our sample satisfying the condition are indeed nonsuicidal. A noted fact is that a value of 1.46 for the “achieve” category is larger than the mean “achieve” values of most genres of writing analyzed using LIWC, as reported in the LIWC documentation [36]. This suggests that relative to others, these individuals’ tweets have a higher proportion of achievement-related words, and that this high degree of achievement talk covaries with nil levels of clinically significant suicidality.

The next node where the tree splits (left branch) contains the “religion” category of LIWC. If an individual’s rate of usage of religion-related words exceeds 0.24, then that individual is labeled as nonsuicidal. As seen above, it is striking that the corresponding leaf node has rather small entropy, giving relatively high confidence to the prediction (90%; 36 of 40). This seems to confirm other studies suggesting that religiosity may act as a protective factor against depression, social isolation, and suicidality. If the rate of religion-related words is low, the prediction of suicidality jumps to just about 50% (12 of 22). The final split of the pruned tree contains the “relativity” category, which is related to notions of motion, space, and time. It provides a rather clean separation between suicidal and nonsuicidal individuals.

## Discussion

### Principal Findings

Suicide continues to be one of the leading causes of death in the US [1] and new methods of assessment capable of tracking suicide risk in real time are required. Our findings reveal that machine learning algorithms can help differentiate between those who are at suicide suicidal risk and those who are not. Below we discuss these findings in light of theories of suicide, implications for public health intervention, and future directions for using social media to reduce suicide.

The notion of using ML approaches to make interpretations of large data has been explored previously. Poulin et al. demonstrated the capacity for an algorithm to identify suicide risk by analyzing clinical notes [48]. Provided the clinical context of the notes, which included specific references to suicidality, their findings may be somewhat expected. Another step beyond, then, is the analysis of data, which were not intended for a professional audience and to identify the user at suicidal risk. It is believed that text in social media includes technical jargon or official diagnoses indicative of suicidal risk. Only a handful of studies have examined measurement of suicidality in social media data using a variety of methodological approaches. One showed that simply tweeting the phrases “want to commit suicide” or “want to die” were predictive of suicidal ideation and behavior [49]. At least two studies have employed machine-learning algorithms to assess suicide risk. One compared level of agreement between humans and algorithms on three categories of suicide risk, finding rates of agreement between 76–80% [22]. Jashinsky and colleagues compared Twitter derived assessment of suicide risk with rates of suicide from the Centers for Disease Control and Prevention (CDC) and showed the correlation between their method and the actual suicidal rates by state across the US [19]. Our study advanced this line of research by validating Twitter data against already validated measures of suicidality at the individual level. This is an important step forward, because the most effective interventions target individuals who are mostly at risk, ideally with an approach that is tailored to their specific needs [50]. Efforts to target individuals at risk would not need to discuss suicide explicitly, but could simply be a directed tweet mentioning, “In moments of crisis, 1-800-273-TALK is a great resource staffed with trained professionals who care.” It is possible that an individual may feel upset at being targeted with such message. However, this is only a speculation and future research could explore tolerance for such messaging approaches and ultimately inform health communication strategies that are nonobtrusive, yet effective.

The fact that tweets including themes of achievement differentiated respondents so well may at first seem surprising in light of theories of suicide. The interpersonal theory of suicide predicts that suicide is most likely when a sense of thwarted belonging and burdensomeness are coupled with an acquired capability for suicide through repeated exposure to painful and provocative experiences [33]. Other prominent theories focus on hopelessness [51] and escape from the self [52]; none include achievement as a key theoretical component. Further, previous research on achievement as a predictor of suicide generally shows no association while controlling depressive symptoms and other common covariates [53-56].

However, it is likely that achievement helps us to rule out suicide rather than to rule it in. Our algorithm appeared to go through two major steps. First, ruling out those who are clearly nonsuicidal (using achievement) and then ruling suicidality in using themes of death and emotional intensity. Traditional assessment of suicide risk has implicitly focused on discerning severe suicidality among populations that often present with thoughts of suicide (eg, individuals seeking treatment for depression or posttraumatic stress disorder); traditional approaches do not typically assess which variables rule out suicide. Our attempt to measure suicidality using social media data in a nonclinical population is distinct from typical methods because it does not ask people to report on symptoms, instead the algorithm monitors a broadcast of comments intended to be shared with anyone who will listen. Hence, achievement likely emerged as a strong differentiating factor because the forward thinking and optimistic nature of achievement is antithetical to suicide. Future research should continue to explore whether a similar “rule out” followed by “ruling in” approach occurs in other machine learning algorithms of social media data.

### Limitations and Strengths

Our study has a number of limitations as well as strengths. First, although self-report measures have an element of socially desirable responding influencing scores, it is possible that social desirability may also play a role in Twitter data. However, when themes predictive of suicide emerge in social media, and thus go against the typical scripts of social media chatter, they could represent a major cry for help that may be more informative than other methods of assessing suicidal risk; we propose that future research should explore this issue. We also encourage examination of other forms of online media (eg, Facebook, blogs, etc) because they may serve a slightly different function than Twitter and thus generate different algorithms for detecting suicide risk. Second, as suicide is a rare event, only limited amounts of clinically significant suicidality was analyzed. Although we cross-validated our own sample, we encourage other researchers to replicate our work in other samples to provide even stronger converging evidence of these machine-learning algorithms. We would especially encourage replication using samples recruited via means other than MTurk, since it is possible that MTurk participants are different from the general population of social media users in ways that influenced the themes we observed in our research (eg, themes of achievement). On the other hand, our study is the first to validate machine-learning algorithms in Twitter data against psychometrically validated measures of suicidality. Moreover, our multimodal assessment of suicidality took place within a sample that is known to be more attentive [57] and representative than college student populations [58], where novel research ideas are often tested. Further, our results provide strong evidence that we are reliably able to differentiate those who are clinically significantly suicidal from those who are not.

### Public Health Significance

Regarding public health approaches to suicide, Twitter offers an unprecedented stream of data connecting individuals to society; our study suggests that there might be a very tangible way we can leverage this phenomenon to do something beneficial. As we further refine our ability to identify suicide risk in real time, our ability to reduce risk for suicide will increase. This may augment existing programs attempting to reduce suicide. For example, suicide hotlines have staff that wait for individuals in crisis to call in; we may enhance these efforts using social media data to proactively identify those who may benefit from their services. When an individual’s public twitter stream indicates clinically significant suicidality, simple interventions such as sending them a private message directing them to 1-800-273-TALK is almost effortless to do, but may have a significant impact. Simple interventions that foster belongingness or connect people to reach-out-and-talk- to-someone resources are likely to help anyone virtually. An important study showed that simply sending follow-up letters to individuals who had been previously hospitalized for suicide or depression reduced the rate of subsequent suicide compared to those who received no such contact[59]. We believe that expanding our portfolio of approaches to include surveillance of social media in order to identify and prevent suicide across the entire population of those who use social media has the potential to substantially reduce the incidence of suicide in the US.

The White House has indicated that suicide prevention is a top priority and has funded a number of initiatives attempting to reduce suicide [60]. However, many attempts to reduce suicide are marked by good intentions but lack a strong empirical base and reach only a limited number of people. In order to extend our reach in a way that can commensurate with the problem of suicide, we need to move beyond status quo approaches that wait for people to seek treatment when they are in deep distress and instead seek them out before they reach the point of crisis.
